# Effect of azithromycin on a red complex polymicrobial biofilm

**DOI:** 10.1080/20002297.2017.1339579

**Published:** 2017-06-16

**Authors:** Hwei Sze Ong, Orit Oettinger-Barak, Stuart G. Dashper, Ivan B. Darby, Kheng H. Tan, Eric C. Reynolds

**Affiliations:** ^a^ Melbourne Dental School, Oral Health Cooperative Research Centre, Bio21 Institute, The University of Melbourne, Carlton, Australia

**Keywords:** Red complex bacteria, azithromycin, biofilm, periodontal disease, antibiotic therapy

## Abstract

Azithromycin has recently gained popularity for the treatment of periodontal disease, despite sparse literature supporting efficiency in treating periodontal bacterial biofilms. The aim of this study was to evaluate the effect of azithromycin on biofilms comprised of *Porphyromonas gingivalis*, *Treponema denticola*, and *Tannerella forsythia* in comparison to an amoxicillin and metronidazole combination. *P. gingivalis* W50, *T. denticola* ATCC35405, and *T. forsythia* ATCC43037 grown under anaerobic conditions at 37°C were aliquoted into 96-well flat-bottom plates in different combinations with addition of azithromycin or amoxicillin + metronidazole at various concentrations. For the biofilm assay, the plates were incubated at 37°C anaerobically for 48 h, after which the biofilms were stained with crystal violet and measured for absorbance at AU_620_. In this model, polymicrobial biofilms of *P. gingivalis *+ *T. denticola*, *P. gingivalis *+ *T. forsythia*, and *T. denticola* + *T. forsythia* were cultured. Combination of all three bacteria enhanced biofilm biomass. Azithromycin demonstrated a minimal biofilm inhibitory concentration (MBIC) of 10.6 mg/L, while the amoxicillin + metronidazole combination was more effective in inhibiting biofilm formation with a MBIC of 1.63 mg/L. Polymicrobial biofilm formation was demonstrated by combination of all three red complex bacteria. Azithromycin was ineffective in preventing biofilm formation within a clinically achievable concentration, whereas the combination of amoxicillin and metronidazole was more effective for this purpose.

## Introduction

Control of subgingival plaque is an essential component of periodontal treatment. While mechanical debridement remains the first line of periodontal treatment [1], not all patients respond favourably [2,3]. Therefore, supplementary antibiotic therapy is recommended in specific cases to improve the treatment outcome [4]. Current knowledge of the susceptibility of oral bacterial biofilms to antimicrobial agents is limited. Although results of studies investigating the effects of antimicrobial agents on oral bacteria have revealed significant differences in bacterial growth in planktonic form compared with biofilm [5–8], most of the research evaluating the effect of antibiotics on oral bacteria have been conducted using planktonic growth [9–14]. As late colonisers in dental biofilm formation [15], the red complex bacteria (*Porphyromonas gingivalis*, *Treponema denticola*, and *Tannerella forsythia*) express synergistic virulence and pathogenicity [16,17]. Among the antibiotics used in treating periodontitis, amoxicillin in combination with metronidazole has been shown to display a strong effect in reducing numbers of non-periodontal bacteria, as well as *P. gingivalis* and *Fusobacterium nucleatum* monomicrobial biofilms *in vitro* [18], and it is currently proposed to be the most clinically and microbiologically advantageous adjunctive antibiotic regime in treating periodontitis [19,20]. Azithromycin, a macrolide antibiotic [21], has also gained popularity for the treatment of periodontitis [22–25]. It is suggested that azithromycin’s pharmacological benefits [26], broad antibacterial spectrum [27], and host modulatory functions [28] make it a viable alternative to the amoxicillin and metronidazole combination. Despite its popularity, there is no literature supporting the efficiency of azithromycin in treating periodontal bacterial biofilms. Therefore, the objective of this study was to evaluate the *in vitro* effect of azithromycin on mono- and polymicrobial biofilm formation comprised of the red complex pathogens *P. gingivalis, T. denticola*, and *T. forsythia* in comparison to the amoxicillin and metronidazole combination.

## Materials and methods

### Bacterial strains, growth medium, and culture conditions

*P. gingivalis* W50, *T. denticola* ATCC® 35405™, and *T. forsythia* ATCC® 43037™ were obtained from the culture collection of the Oral Health Cooperative Research Centre, Melbourne Dental School, The University of Melbourne. Planktonic bacterial cultures of *P. gingivalis, T. denticola*, and *T. forsythia* were routinely grown in oral bacteria growth medium (chemicals supplied by Sigma–Aldrich, and growth media by Oxoid Australia), a modified and adapted version of new oral spirochete medium [29], and GM-1 [30,31], which had been pre-reduced under anaerobic conditions. The cultures were maintained in an anaerobic workstation (MG500; Don Whitley Scientific) at 37°C. Growth was monitored by measuring absorbance at a wavelength of 650 nm (AU_650_), and *P. gingivalis* and *T. forsythia* were harvested during the mid-exponential phase at an AU_650_ of 0.6, which equates to a cell density of ~1.5 × 10^9^ cells/mL [32]. *T. denticola* was grown to an AU_650_ of 0.15, which equates to a cell density of ~1.0 × 10^8^ cells/mL. Culture purity was routinely monitored by Gram staining and colony morphology examination under light microscope.

### Effects of antibiotics on planktonic polymicrobial culture

Exponentially growing *P. gingivalis* Pg) and *T. forsythia* Tf) cells diluted to an AU_650_ of 0.15 and undiluted *T. denticola* at the same AU_650_ were used as inoculum. Two hundred microliters of *P. gingivalis*, *T. denticola*, or *T. forsythia* as a monospecies inoculum and the combination of each two bacterial species at equal volumes (100 µL each), as well as all three species (67 µL each) as a polymicrobial inoculum, were aliquoted into 96-well flat-bottom plates (Nunc; Thermo Scientific) to provide the same total number of bacterial cells per inoculum. Azithromycin, amoxicillin, and metronidazole (Thermo Multiskan Ascent; Pathtech) were dissolved in deionized water (MQ). Dissolved metronidazole and amoxicillin were mixed in a 1:1 ratio. Stock solutions at 100 mg/L of azithromycin and amoxicillin + metronidazole (1:1 ratio) were diluted in MQ of different volumes to achieve final antibiotic concentrations in the range of 0.01–100 mg/L.Twenty microliters of each antibiotic concentration was added into the wells of a 96-well plate containing the bacterial cultures. Native bacterial growth with no antibiotic added, as well as uncultured growth medium, served as controls. The plate was sealed with microtiter plate film to maintain the anaerobic condition and was incubated at 37°C, with periodic shaking to prevent bacterial cell precipitation. Growth was monitored for 48 h by measuring absorbance at a wavelength of 620 nm (AU_620_) using a microplate reader (Thermo Multiskan Ascent; Pathtech). The minimal inhibitory concentration (MIC) for the antibiotics was calculated by linear regression.

### Effects of antibiotics on mono- and polymicrobial biofilm formation

Exponentially growing *P. gingivalis* and *T. forsythia* cells diluted to an AU_650_ of 0.15 and undiluted *T. denticola* at the same AU_650_ were used as inoculum. Two hundred microliters of *P. gingivalis*, *T. denticola*, *T. forsythia*, *P. gingivalis + T. denticola*, *P. gingivalis + T. forsythia*, *T. denticola + T. forsythia*, and *P. gingivalis + T. denticola + T. forsythia*bacterial cultures in equal volumes were aliquoted into 96-well flat-bottom plates. Antibiotic dilution, concentrations, and volume used were similar to the planktonic assay. Plates were sealed and incubated at 37°C anaerobically for 48 h.

### Crystal violet staining for biofilm assay

Crystal violet staining assay was adapted and modified from Dashper et al. [33]. The adherent biofilms were rinsed with 200 μL of MQ and incubated with 0.1% crystal violet. The crystal violet stained biofilms were then dissolved in 80% ethanol + 20% acetone through repeated pipetting before transfer to a new 96-well plate. Quantification of the biofilms was carried out by measuring AU_620_ using a plate reader (Perkin Elmer Wallac VICTOR1420 Multilabel Counter; PerkinElmer, Inc.). The minimal biofilm inhibitory concentration (MBIC) for the polymicrobial biofilm was calculated by linear regression.

### Statistical analyses

For each bacterial combination and antibiotics concentration, biofilm formation in the presence of azithromycin was compared to that in the presence of amoxicillin and metronidazole using Student’s *t-*test. The significance level was set at 5%.

## Results

### Susceptibility of planktonic polymicrobial culture to antibiotics

Azithromycin and the combination of amoxicillin + metronidazole were evaluated to determine the MIC of the planktonic polymicrobial culture. Azithromycin and the combination of amoxicillin + metronidazole had a MIC of 1.52 mg/L and 0.17 mg/L, respectively (Table 1).

**Table 1.** The MIC and MBIC of azithromycin and amoxicillin + metronidazole (1:1 ratio) against polymicrobial planktonic cells and biofilms determined using the 96-well plate model

Antibiotic	MIC (mg/L)	MBIC (mg/L)
Azithromycin	1.52 (*R* = 0.988)	10.6 (*R* = 0.639)
Amoxicillin + metronidazole (1:1)	0.17 (*R* = 0.993)	1.63 (*R* = 0.940)

Both MIC and MBIC were determined by linear regression using growth data from a minimum of three biological replicates. *R* = correlation coefficient.

MIC, minimal inhibitory concentration; MBIC, minimal biofilm inhibitory concentration.

### Biofilm formation

**Figure 1.**
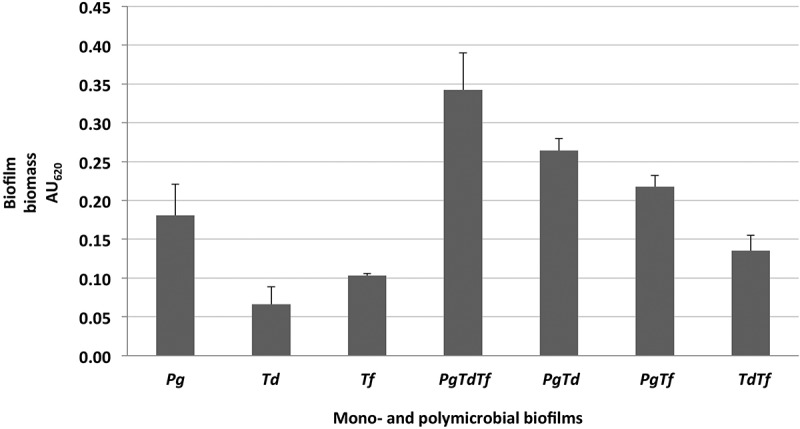
Formation of mono- and polymicrobial biofilms in a 96-well plate model after 48 h of incubation at 37°C under anaerobic condition. Native bacterial growth with addition of uncultured growth medium and no antibiotic served as controls. Adherent biofilms were stained with 0.1% crystal violet and the optical density at AU_620_ was measured. Data represent the mean AU_620_ value of a minimum of three biological replicates.

In this model, polymicrobial biofilm formation between *P. gingivalis *+ *T. denticola*, *P. gingivalis* + *T. forsythia*, as well as *T. denticola *+ *T. forsythia* was demonstrated (Figure 1). The *P. gingivalis* and *T. forsythia* combination enhanced biofilm formation but not as much as that of *P. gingivalis* and *T. denticola*. The combination of *P. gingivalis*, *T. denticola*, and *T. forsythia* formed the most biofilm (AU_620_ = 0.34 ± 0.05; Figure 1). Whenever *P. gingivalis* was involved, the biofilm had a tendency to establish better. *T. denticola* (AU_620_ = 0.07 ± 0.02) and *T. forsythia* (AU_620_ = 0.10 ± 0.00) formed minimal biofilm when cultured on their own, particularly *T. denticola*.

### Susceptibility of mono- and polymicrobial biofilms to antibiotics

**Figure 2.**
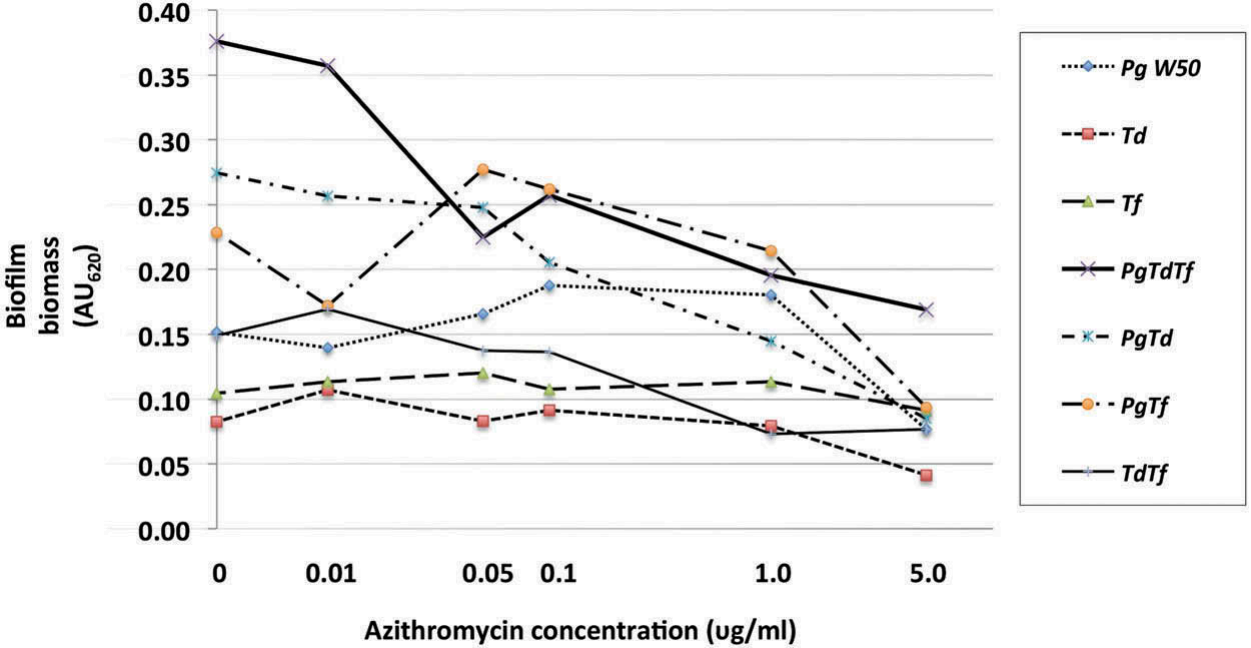
Effect of azithromycin up to 5.0 mg/L on the red complex mono- and polymicrobial biofilms in a 96-well plate model. Azithromycin at concentrations 0–100 mg/L was incubated with bacterial cultures for 48 h under anaerobic conditions. Data points represent the mean AU_620_ value of a minimum of three biological replicates. Note the categorical scale.

**Figure 3.**
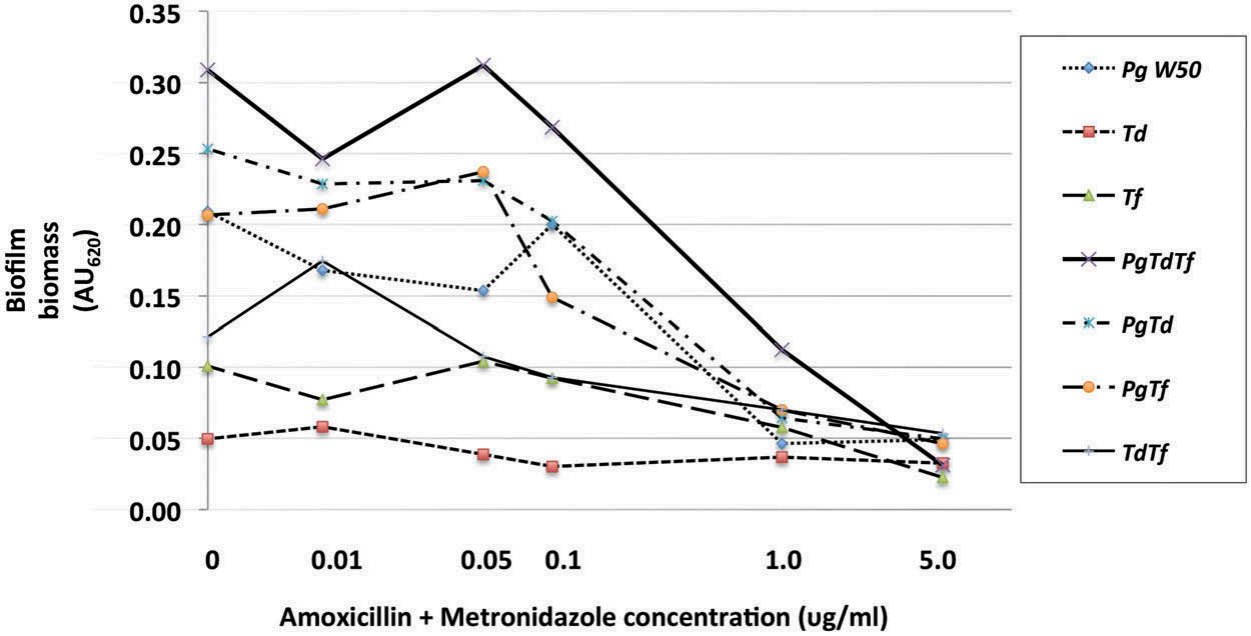
Effect of amoxicillin + metronidazole up to 5.0 mg/L on the red complex mono- and polymicrobial biofilms in a 96-well plate model. Amoxicillin + metronidazole in a 1:1 ratio at concentrations 0–100 mg/L was incubated with bacterial cultures for 48 h at 37°C anaerobically. Data points represent the mean AU_620_ value of a minimum of three biological replicates. Note the categorical scale.

**Figure 4.**
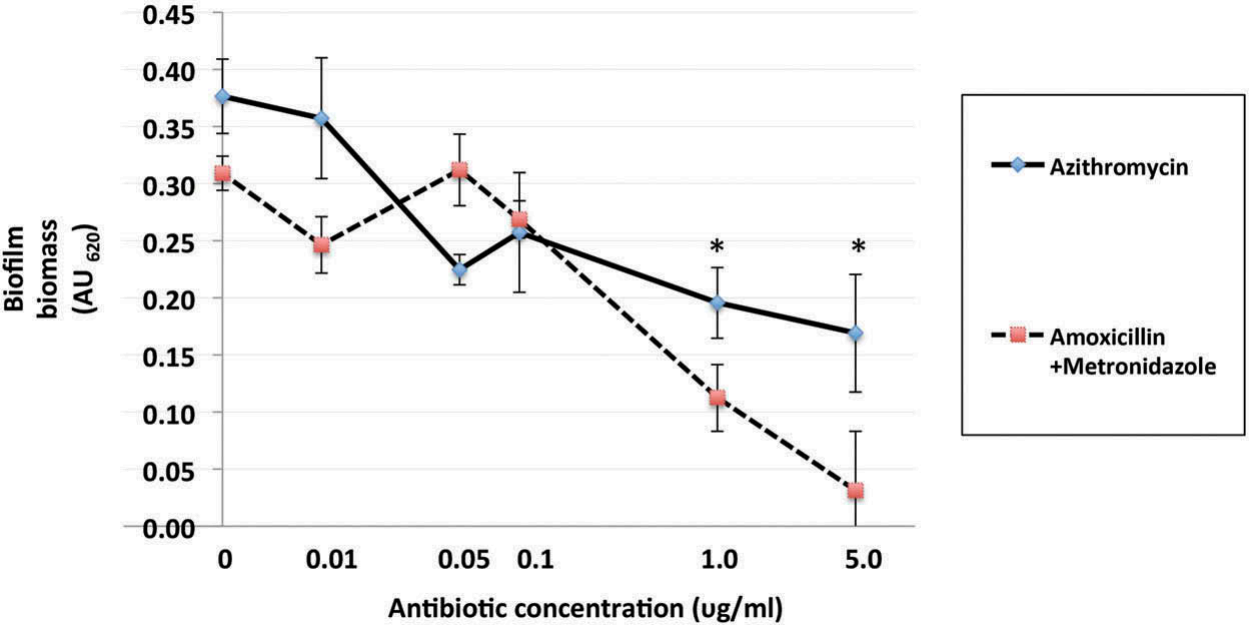
Effects of azithromycin and amoxicillin + metronidazole (1:1 ratio) up to 5.0 mg/L on formation polymicrobial biofilms after 48 h of anaerobic incubation at 37°C in a 96-well plate model. Azithromycin and amoxicillin + metronidazole (1:1 ratio) at concentrations 0–100 mg/L were incubated with bacterial cultures. Data points represent the mean AU_620_ value of a minimum of three biological replicates and the standard deviation. *p < 0.05, Student’s paired *t*-test.

The effect of azithromycin and amoxicillin + metronidazole on mono- and polymicrobial biofilm formation varied, with amoxicillin + metronidazole being more efficacious than azithromycin (Figures 2 and 3). The combination of amoxicillin + metronidazole at a concentration of 1.0 mg/L reduced the biomass of *P. gingivalis* monomicrobial biofilms by 78%. Concentrations of amoxicillin + metronidazole at 1.0 mg/L and 5.0 mg/L reduced the polymicrobial biofilm biomass by 64 and 89%, respectively(Figure 4), which was significantly better than azithromycin’s effect of 48 and 55% reduction for those concentrations (*p* < 0.05). The amoxicillin + metronidazole combination effect was most pronounced in cultures involving *P. gingivalis*. Of the antibiotics tested, amoxicillin + metronidazole was the most efficacious, with a MBIC against the polymicrobial biofilm of 1.63 mg/L, while azithromycin was much less effective, with a MBIC of 10.6 mg/L (Table 1).

## Discussion

In this *in vitro* study, three-species polymicrobial biofilms of the red complex bacteria yielded more biofilm biomass compared to monospecies or two-species biofilms. *P. gingivalis*, in particular, seemed to increase the biofilm biomass. The red complex bacteria appear later in biofilm development [15] and are repeatedly found together in high levels in the subgingival biofilms of subjects with periodontitis [34]. Although these three species do not fully represent the complexity of the polymicrobial biofilms associated with a pathogenic subgingival plaque, they do form an interdependent bacterial community near the gingival epithelium, and the emergence of this community is associated with disease severity and progression [16,35,36]. Consistent with the current findings, *T. denticola* strains are known to form insignificant amounts of biofilm when incubated on inert surfaces *in vitro* [37], while *P. gingivalis* is able to form substantial biofilms *in vitro* [38]. Also consistent with the current findings, a positive cooperativity between *T. denticola* and *P. gingivalis* in biofilm formation has been demonstrated [39]. The two species co-aggregate [40] and exhibit a mutualistic enhancement of growth *in vitro*, with each producing nutrients that stimulate the growth of the other [41]. Similarly, *T. forsythia* also accumulates better in dual species biofilms involving *T. denticola* [42] or *F. nucleatum* [43]. Cell extracts of *T. forsythia* have been shown to stimulate the growth of *P. gingivalis* [44]. Furthermore, *T. forsythia* has been detected more frequently and in greater numbers in deep periodontal pockets containing *P. gingivalis* [45]. In an earlier study using the same methodology with real-time polymerase chain reaction enumeration of the individual bacterial species, it was demonstrated that all three species were present in the 48 h model biofilms, with *T. denticola* representing 66% of the total cells present and *P. gingivalis* and *T. forsythia* contributing18% and 16% of the total biofilm cells, respectively [46].

Of the antibiotics examined in this study, the amoxicillin and metronidazole combination produced the best results, inhibiting both *in vitro* planktonic and biofilm growth of the polymicrobial combination at relatively low concentrations compared to azithromycin. There are few other studies examining the effects of antibiotics on polymicrobial oral bacterial biofilms. Belibasakis and Thurnheer [18] recently reported that amoxicillin + metronidazole (1:1 ratio) at a concentration of 15 mg/L caused reductions to total cell numbers of established 10-species polymicrobial biofilms, significantly reducing *P. gingivalis* numbers in these biofilms after 24 h of exposure. Soares et al. have recently reported that the amoxicillin + metronidazole combination significantly reduced metabolic activity of 35 subgingival bacterial species residing in complex biofilms [47]. The present study determined that the MBIC of the amoxicillin + metronidazole combination was 1.63 mg/L for the prevention of polymicrobial biofilm establishment, which should be clinically achievable. Amoxicillin concentrations in gingival crevicular fluid have been shown to reach up to 13–14 mg/L [48] and 13 mg/L for metronidazole [49] following a 500 mg per oral dose. The total bacterial load of *P. gingivalis*, however, might be considerably lower in *in vivo* biofilms compared to those reported here, thereby underestimating the clinical efficacy.

Very few studies involving red complex bacteria and azithromycin have been conducted, despite the increasingly widespread clinical use of the antibiotic [50,51]. Macrolides have been found to reduce bacterial adhesion, resulting in reduced biofilm formation, even at very low concentrations in a dose-dependent relationship [52]. *In vitro* model studies have reported that azithromycin decreased metabolic activity, biofilm viability, and density of *P. gingivalis* at sub-MIC levels of approximately 0.1 mg/L [53,54]. To date, there are no studies reporting the MBIC for azithromycin against polymicrobial biofilms involving the red complex bacteria. Azithromycin concentrations in gingival crevicular fluid have been shown to reach up to 7–8 mg/L [55,56] following a 500 mg oral dose, and even lower values in periodontal tissues [22]. The azithromycin MBIC of 10.6 mg/L against the polymicrobial biofilm formation obtained in this study is almost 10-fold higher than the MBIC of the amoxicillin + metronidazole combination and is likely to be clinically unachievable. Furthermore, it has been suggested that *P. gingivalis* and *T. forsythia* may be developing resistance to azithromycin [57], and resistant *T. forsythia* has been isolated from patients with untreated periodontitis [58].

Mechanical debridement of the subgingival plaque biofilm is the first line of treatment for periodontitis [59–61], and antibiotic supplementation is warranted in certain cases [62,63]. The emergence of high bacterial resistance [64,65] and tolerance [66] to antimicrobials has led to the recommendation that these agents should only be used in conjunction with mechanical debridement in cases where there is a need to improve the treatment outcome. The biofilm assay model described in this study involved a short exposure (48 h) of the red complex bacteria to the antimicrobial agent during biofilm formation compared to previous studies where the antimicrobial agents were tested on established biofilms [67,68]. This was done to mimic the clinical situation following mechanical debridement before the bacteria have had time to reform an established mature biofilm. In this model, azithromycin was shown to be ineffective, whereas the amoxicillin and metronidazole combination was far more effective in preventing polymicrobial biofilm formation. Mechanical removal of the subgingival plaque biofilm in conjunction with the amoxicillin and metronidazole combination protocol may therefore enhance treatment outcomes.
